# Identification of symptomatic carotid artery plaque: a predictive model combining angiography with optical coherence tomography

**DOI:** 10.3389/fneur.2024.1445227

**Published:** 2024-08-30

**Authors:** Jun Zhuo, Lin Wang, Ruolin Li, Zhiyuan Li, Junhu Zhang, Yunjian Xu

**Affiliations:** ^1^Medical Engineering and Technology Research Center, School of Radiology, Shandong First Medical University and Shandong Academy of Medical Sciences, Taian, China; ^2^Medical Science and Technology Innovation Center, Institute of Medical Engineering and Interdisciplinary Research, Shandong First Medical University and Shandong Academy of Medical Sciences, Jinan, China; ^3^Department of Interventional Radiology, Affiliated Hospital of Jining Medical University, Jining, China; ^4^Department of Neurology, Affiliated Hospital of Jining Medical University, Jining, China

**Keywords:** digital subtraction angiography, optical coherence tomography, symptomatic carotid plaque, plaque surface morphology, vulnerable plaque

## Abstract

**Objective:**

Symptomatic carotid artery disease is indicative of an elevated likelihood of experiencing a subsequent stroke, with the morphology of plaque and its specific features being closely linked to the risk of stroke occurrence. Our study based on the characteristics of carotid plaque assessed by optical coherence tomography (OCT), the plaque morphology evaluated by digital subtraction angiography (DSA) and clinical laboratory indicators were combined, develop a combined predictive model to identify symptomatic carotid plaque.

**Methods:**

Patients diagnosed with carotid atherosclerotic stenosis who underwent whole-brain DSA and OCT examination at the Affiliated Hospital of Jining Medical University from January 2021 to November 2023 were evaluated. Clinical features, as well as DSA and OCT plaque characteristics, were analyzed for differences between symptomatic and asymptomatic cohorts. An analysis of logistic regression was carried out to identify factors associated with the presence of symptomatic carotid plaque. A multivariate binary logistic regression equation was established with the odds ratio (OR) serving as the risk assessment parameter. The receiver operating characteristic curve was utilized to assess the combined predictive model and independent influencing factors.

**Results:**

A total of 52 patients were included in the study (symptomatic: 44.2%, asymptomatic: 55.8%). Symptomatic carotid stenosis was significantly linked to four main factors: low-density lipoprotein-cholesterol >3.36 mmol/L [OR, 6.400; 95% confidence interval (CI), 1.067–38.402; *p* = 0.042], irregular plaque (OR, 6.054; 95% CI, 1.016–36.083; *p* = 0.048), ruptured plaque (OR, 6.077; 95% CI, 1.046–35.298; *p* = 0.048), and thrombus (OR, 6.773; 95% CI, 1.194–38.433; *p* = 0.044). The combined predictive model generated using four indicators showed good discrimination (Area Under Curve, 0.924; 95% CI, 0.815–0. 979). The *p* value was <0.05 with 78.26% sensitivity and 93.10% specificity.

**Conclusion:**

OCT is valuable in evaluating the plaque characteristics of carotid atherosclerotic stenosis. The combined predictive model comprising low-density lipoprotein-cholesterol >3.36 mmol/L, irregular plaque, ruptured plaque, and thrombus could help in the detection of symptomatic carotid plaque. Further research conducted on additional independent cohorts is necessary to confirm the clinical significance of the predictive model for symptomatic carotid plaque.

## 1 Introduction

Carotid atherosclerotic stenosis is a significant factor in causing ischemic stroke (IS) and transient ischemic attack (1, 2). The development of carotid artery stenosis (CAS) or occlusion due to plaque formation is the primary mechanism underlying IS, leading to reduced blood flow (hypoperfusion) (3, 4). Additionally, plaque detachment can result in artery–artery embolism, further contributing to the risk of IS (3). Numerous studies have demonstrated a positive correlation between CAS severity and heightened risk of IS (5). For example, the risk of IS in patients with ≥50% carotid stenosis is about 2.5 times higher than that in patients with <50% stenosis (3). The incidence of symptomatic CAS escalates with advancing age and is believed to confer a greater propensity for recurrent stroke in contrast to asymptomatic CAS (6, 7). Research findings indicate that approximately two-thirds of individuals diagnosed with carotid atherosclerosis, a condition associated with stroke, do not exhibit any symptoms prior to its onset (8). Furthermore, there is no straightforward correlation between symptom severity and the extent of vascular stenosis (9). Therefore, the degree of CAS does not serve as a reliable indicator of the risk of experiencing a stroke. A growing body of clinical evidence suggests a relationship between the morphological features of plaque, its susceptibility to rupture, and the manifestation of patient symptoms as well as the risk of stroke (10, 11).

In addition to digital subtraction angiography (DSA), which is widely regarded as the benchmark technique for diagnosing carotid stenosis in numerous randomized trials, various frequently employed approaches exist for detecting carotid plaque (9, 12). Some of the imaging techniques used are ultrasound, computed tomography, and magnetic resonance imaging (13). However, their ability to accurately identify plaque morphology is limited due to the low resolution. Optical coherence tomography (OCT) is an emerging endovascular imaging modality that employs near-infrared light to produce high-resolution (15–20 μm) images of endovascular structures (14). This technique enables a comprehensive assessment of plaque composition *in vivo* and has been substantiated through histological controls (14, 15). However, prior research investigating the prognostic significance of plaque imaging in individuals with both symptomatic and asymptomatic plaques has not integrated clinical variables with multiple imaging biomarkers (16, 17).

The current research sought to assess plaque characteristics utilizing OCT and to develop a combined predictive model comprising clinical indicators, plaque surface morphology from DSA, and plaque features from OCT to enhance the identification of symptomatic carotid plaque.

## 2 Methods

### 2.1 Study population

A total of 59 non-consecutive patients diagnosed with CAS at the Affiliated Hospital of Jining Medical University (Jining, China) between January 2021 and November 2023 underwent a DSA examination and OCT study. Informed consent was obtained from all patients for the procedures conducted, and a retrospective case review was authorized by the Ethics Committee of the Affiliated Hospital of Jining Medical University. Indications for DSA examinations included patients who required further identification of the degree of vascular stenosis and/or carotid stent implantation. Indications for OCT studies included vascular stenosis in patients needing additional assessment of the nature of stenosis lesion and plaque characteristics.

Patients in the study were separated into two groups according to the presence or absence of prior symptoms, namely symptomatic and asymptomatic groups. Patients in the symptomatic cohort presented with a documented medical history of transient ischemic attack, amaurosis fugax, or stroke in the vascular territory supplied by the internal carotid artery (ICA) within the 6 months prior the examination (16). Patients who met the following criteria were excluded: (1) Complications of myocardial infarction, cardiogenic shock, contrast-media allergy, renal insufficiency, or cerebral tumor; (2) OCT imaging catheter could not pass through CAS or severe tortuosity segment (C-shaped or S-shaped segment of the internal carotid artery C1 is considered tortuous); (3) Inability to perform analysis due to incomplete baseline or imaging data; (4) Poor OCT image quality for interpretation due to non-ideal vascular blood clearance; (5) The imaging experts disagreed on the interpretation of the images and could not come to a consensus after discussing. Two cases had unsatisfactory blood clearance, the inability of the OCT catheter to navigate through the lesion in three cases due to vascular tortuosity, OCT images of two patients could not meet the diagnostic requirements. A total of 52 patients were enrolled in the study.

### 2.2 Data collection

Clinical information on demographic data (age, sex), vascular risk factors (hypertension, hyperlipidemia, diabetes, coronary heart disease, and smoking history), laboratory tests (glucose, total cholesterol (TC), triglycerides, high-density lipoprotein-cholesterol (HDL-C), low-density lipoprotein-cholesterol (LDL-C), very low-density lipoprotein-cholesterol (VLDL-C), and homocysteine), and previous medication history (antihypertensive, antiplatelet, lipid-lowing, and hypoglycemic drugs) were collected from clinical records. DSA and OCT imaging data were also recorded.

### 2.3 DSA image acquisition and analysis

All patients underwent standard whole-brain DSA interventional procedures via the femoral approach. Heparin (50,000 U) was used for anticoagulation before the operation. Angiographic images were transmitted to a CV-NET workstation (Beijing Sichuang Company, Beijing, China) for vascular stenosis analysis and stenosis length measurement, which were separately carried out and averaged by two professional technicians. DSA imaging findings, including the surface morphology of carotid plaques (smooth and irregular) and stenosis type (concentric and eccentric). Plaques were considered as irregular if there has irregular dilatation (18). Plaques were considered smooth if angiographic images did not show any significant surface irregularities (19).

The results were meticulously documented and individually assessed by three seasoned neuro-interventional specialists. In cases where differing interpretations arose, a thorough discussion ensued until a unanimous conclusion was reached. In instances where consensus could not be achieved, enrollment was canceled.

### 2.4 OCT image acquisition and analysis

OCT was based on the standard operation procedure recommended by China’s OCT technology experts. OCT equipment (OPTISTM Mobile, Abbott Medical Supplies Co, Shanghai, China) and OCT imaging catheters (DragonFly™ intravascular imaging catheter, Abbott Medical Supplies Co, Shanghai, China) were used for OCT evaluation. DSA was used to define the carotid artery lesions. Then, distal embolic protection device (Abbott Medical Supplies Co., Shanghai, China) was inserted over the distal cervical segment of the ICA in patients with moderate–severe stenosis. Transend™ microguidewire (Boston Scientific Corp., Natick, MA, United States) was inserted over the distal cervical segment of the ICA in patients with mild stenosis. Slowly, the OCT catheter was inserted over the guidewire and moved beyond the ICA lesion. An OCT image was obtained by injecting 18 mL of undiluted iodixanol 32 (GE Healthcare Ireland Limited, County Cork, Ireland) at a flow rate of 8 mL/s through the guiding catheter. Calibration of images was done through Z-offset adjustment. Automatic pullbacks covered 75 mm of the vessel at a velocity of 180 frames/s.

**Figure 1:**
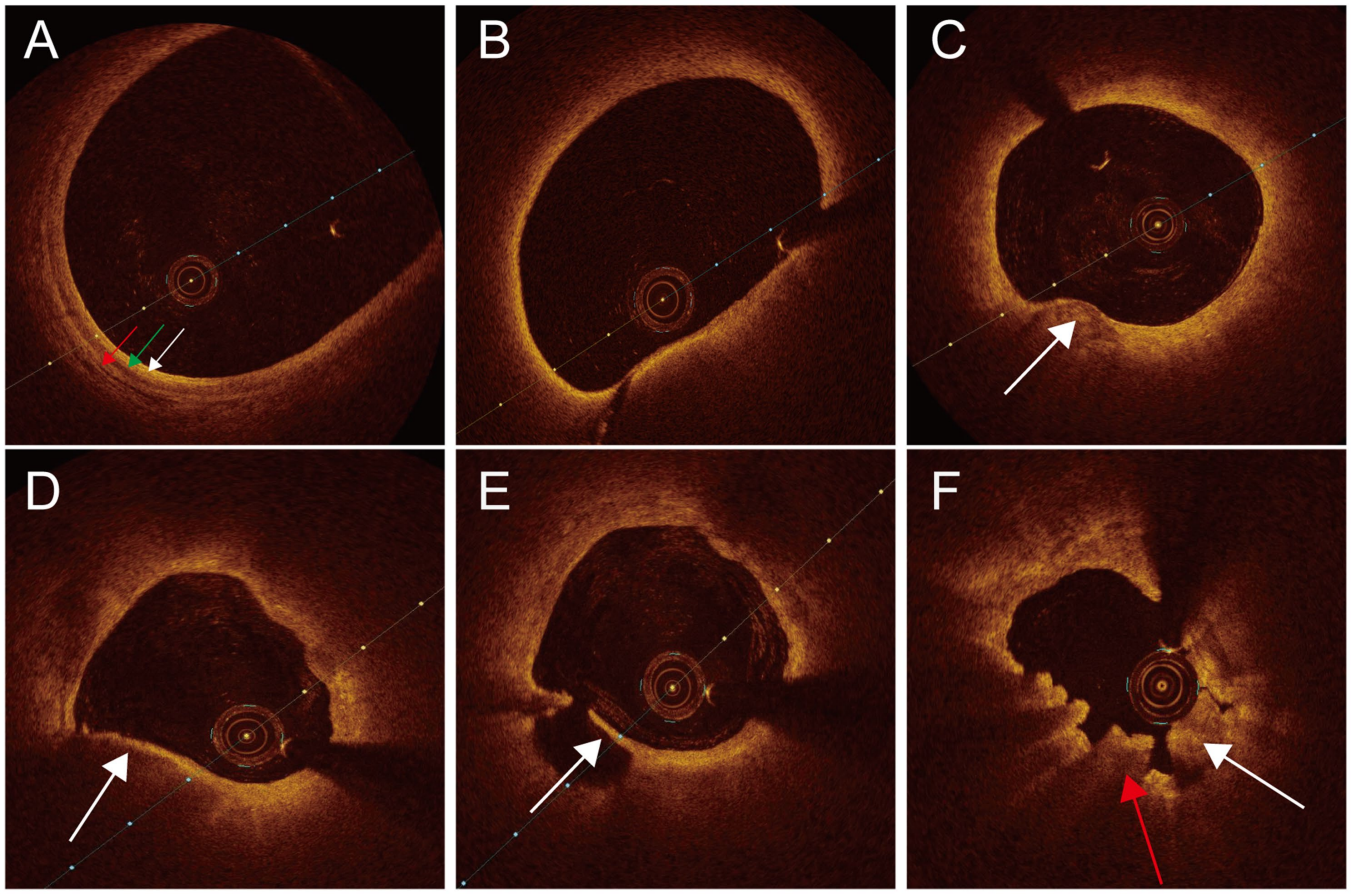
Normal and pathological OCT images of carotid arteries. **(A)** Normal OCT image intima (white arrow), tunica media (green arrow), adventitia (red arrow). **(B)** TCFA. **(C)** Calcified plaque (white arrow). **(D)** Lipid plaque (white arrow). **(E)** Plaque rupture (white arrow). **(F)** Thrombus white thrombus (white arrow), red thrombus (red arrow).

Three experienced observers conducted OCT image analysis while blinded to the clinical data. Established criteria for OCT plaque characterization were followed to ensure all observers unanimous results. If there were differing opinions, they discussed until reaching a unanimous conclusion. If consensus could not be reached, enrollment was canceled. The structure of the three membranes (intima, media, and adventitia) in the normal OCT images was clear, and vascular blood clearance was ideal. Pathological OCT images had the following characteristics: (1) Thin-cap fibroatheroma (TCFA) was characterized by a plaque exhibiting a maximal lipid arc exceeding 180° and a fibrous cap thickness of 65 μm or less; (2) Plaque rupture was identified as a discontinuous fibrous cap and (or) a ruptured cavity; (3) Lipid plaque: strong attenuation areas, low signal, blurred edge; (4) Calcified plaque: low signal or heterogeneous areas with sharp edges; (5) Red thrombus: manifested as weak signal, high back reflex with shadow image; (6) White thrombus: manifested as strong signal, low back reflection image with few shadows (Figure 1).

### 2.5 Statistical analysis

SPSS26.0 (IBM, Armonk, New York, United States) was used for data analysis in this experiment. The counting data were described in the form of frequency (percentage %), and Chi-square analysis (Fisher exact probability method) was used to analyze the counting data. The measurement data that follow normal or approximate normal distribution are described by means and standard deviation, and analyzed by independent sample t test. For the data that do not follow the normal distribution, the median (upper quartile, lower quartile) is used to describe, and the non-parametric test (U test) is used to analyze the difference. With OR without symptoms as the dependent variable (1 for symptomatic group and 0 for asymptomatic group), variables with *p* values less than 0.1 in the univariate analysis were included in the subsequent multivariate logistic regression analysis. The counting data (i.e., categorical variables) were selected and appropriate reference layers were used, and OR was used as the risk assessment parameter. Multivariate binary logistic regression equation was established, then a four-index joint prediction model was carried out, and the ROC curve was used to analyze the joint prediction model and independent influencing factors. In this study, *p* < 0.05 was considered to have statistical significance, and this study was conducted by bilateral tests.

## 3 Results

### 3.1 Patient characteristics in symptomatic and asymptomatic groups

Fifty two patients (36 males, 69.2%) with an average age of 63.38 years were included. Twenty-three patients (44.2%) were categorized into the symptomatic group and 29 patients (55.8%) into the asymptomatic group. When compared to asymptomatic group, symptomatic patients had elevated LDL-C levels (3.85 mmol/L vs. 2.03 mmol/L, *p* = 0.001). No notable variation was observed in additional clinical features among symptomatic and asymptomatic groups (*p* > 0.05) (Table 1).

**Table 1:** Demographic and clinical data of the study population.

Variable	Group		Total (*n* = 52)	*t*/χ^2^/*Z*	*p-*value
	Asymptomatic (*n* = 29)	Symptomatic (*n* = 23)			
Age (years)	64.31 ± 9.50	62.22 ± 7.12	63.38 ± 8.51	0.879	0.384
Male	23 (79.3)	13 (56.5)	36 (69.2)	3.127	0.077
**Clinical features**					
Hypertension	16 (55.2)	11 (47.8)	27 (51.9)	0.277	0.598
Diabetes	17 (58.6)	16 (69.6)	33 (63.5)	0.663	0.416
Hyperlipidemia	19 (65.5)	12 (52.2)	31 (59.6)	0.949	0.330
Coronary heart disease	15 (51.7)	12 (52.2)	27 (51.9)	0.001	0.974
Smoking history	18 (62.1)	14 (60.9)	32 (61.5)	0.008	0.930
**Current medications**					
Aspirin	15 (51.7)	13 (56.5)	28 (53.8)	0.119	0.730
Clopidogrel	11 (37.9)	8 (34.8)	19 (36.5)	0.055	0.815
Statin	13 (44.8)	12 (52.2)	25 (48.1)	0.277	0.598
**Lab test**					
Glucose (mmol/L)	5.32 ± 1.33	5.35 ± 0.73	5.33 ± 1.09	−0.094	0.925
Total cholesterol (mmol/L)	4.04 (3.38, 4.59)	4.33 (3.56, 5.11)	4.21 (3.49, 4.82)	−1.244	0.214
Triglycerides (mmol/L)	1.28 (0.86, 1.71)	1.50 (0.89, 2.35)	1.34 (0.87, 1.91)	−1.483	0.138
HDL-C (mmol/L)	1.19 (0.96, 1.34)	1.13 (1.05, 1.56)	1.17 (1.00, 1.38)	−0.415	0.678
LDL-C (mmol/L)	2.03 (1.71, 3.06)	3.85 (3.01, 4.17)	2.97 (1.92, 3.92)	−3.961	0.001
VHDL-C (mmol/L)	0.27 (0.22, 0.42)	0.28 (0.25, 0.33)	0.28 (0.23, 0.35)	−0.046	0.963
homocysteine (μmol/L)	11.18 ± 2.48	11.00 ± 2.57	11.10 ± 2.49	0.254	0.801

Data were expressed as median (interquartile range) or number (percentage) as appropriate. HDL-C, high-density lipoprotein-cholesterol; LDL-C, low-density lipoprotein-cholesterol; VLDL-C, very low-density lipoprotein-cholesterol.

### 3.2 Contrasting carotid plaque features between symptomatic and asymptomatic groups

Comparison of carotid plaque features between symptomatic and asymptomatic groups was evaluated using DSA and OCT (Table 2). The symptomatic group showed more irregular plaque surface morphology compared to that in the asymptomatic group (78.3% vs. 31.0%, *p* = 0.001). Plaques were also more likely to be rupture in symptomatic patients than in asymptomatic patients (73.9% vs. 17.2%, *p* = 0.001). Symptomatic patients displayed higher rates of thrombus formation compared to asymptomatic group (78.3% vs. 17.2%, *p* = 0.001). No notable variation could be observed in other plaque features (*p* > 0.05).

**Table 2:** Plaque characteristics in the symptomatic and asymptomatic groups.

Variable	Group		Total (*n* = 52)	*t*/χ^2^/*Z*	*p-*value
	Asymptomatic (*n* = 29)	Symptomatic (*n* = 23)			
**DSA**					
Diameter stenosis rate	54.73 ± 8.59	54.37 ± 15.53	54.57 ± 12.02	0.106	0.916
Stenosis length (mm)	22.71 ± 2.88	21.82 ± 2.61	22.32 ± 2.77	1.161	0.251
Irregular	9 (31.0)	18 (78.3)	27 (51.9)	11.460	0.001
**OCT**					
Lipid plaque	17 (58.6)	17 (73.9)	34 (65.4)	1.325	0.250
Fibrous plaque	13 (44.8)	9 (39.1)	22 (42.3)	0.171	0.680
Calcified plaque	10 (34.5)	5 (21.7)	15 (28.8)	1.015	0.314
TCFA	11 (37.9)	12 (52.2)	23 (44.2)	1.055	0.304
Macrophage accumulation	8 (27.6)	10 (43.5)	18 (34.6)	1.431	0.232
Cholesterol crystals	7 (24.1)	6 (26.1)	13 (25)	0.026	0.872
Plaque rupture	5 (17.2)	17 (73.9)	22 (42.3)	16.878	0.001
Thrombus	5 (17.2)	18 (78.3)	23 (44.2)	19.362	0.001

Data were expressed as numbers (percentages) as appropriate. DSA, digital subtraction angiography; OCT, optical coherence tomography; TCFA, thin-cap fibroatheroma.

### 3.3 Prediction model combining clinical and imaging indicators to identify symptomatic CAS

A logistic regression analysis was conducted, and the presence/absence of symptoms as the dependent variable and *p* < 0.1 as the explanatory variable.

**Figure 2:**
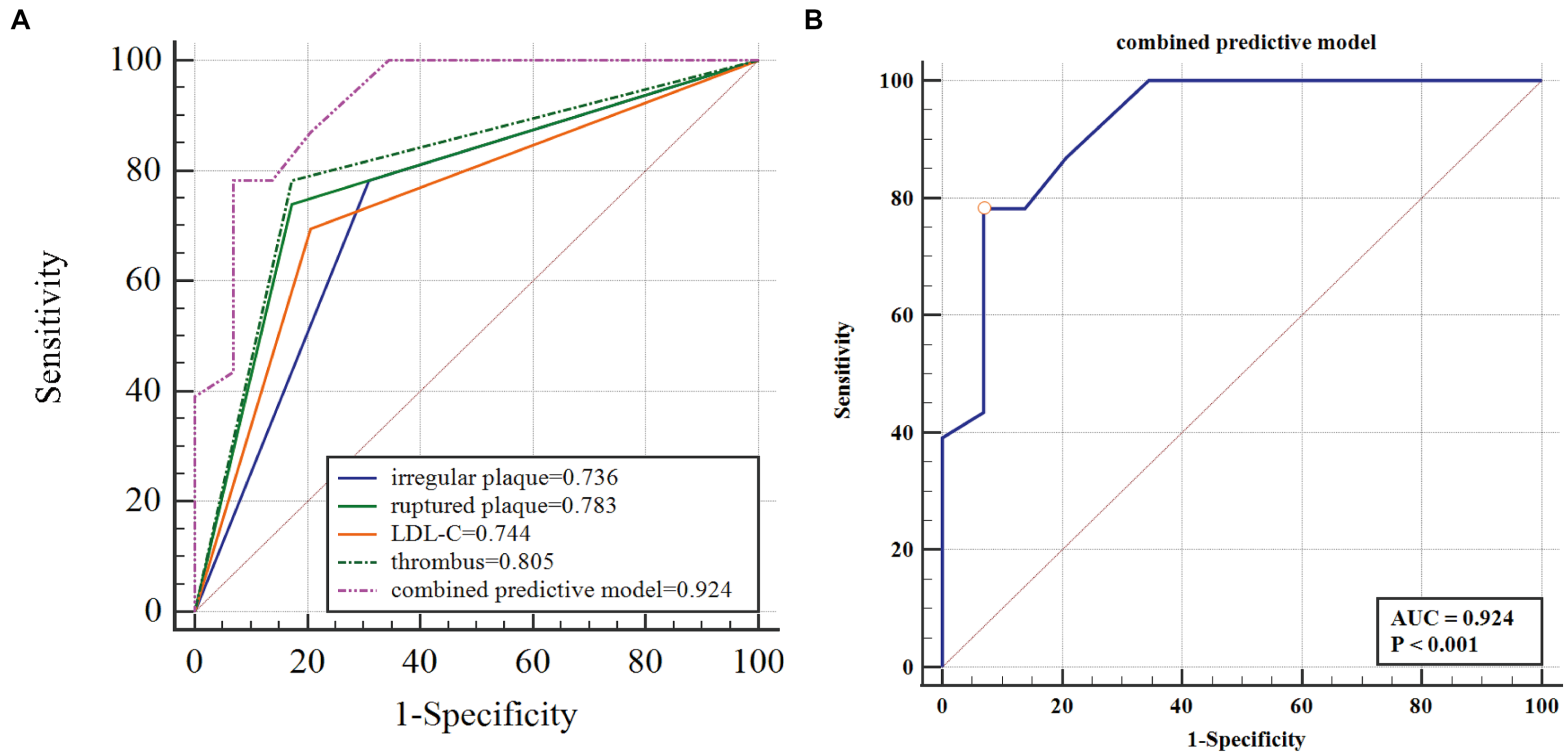
The four-item factor of symptomatic carotid plaque and the ROC curve. LDL-C, low density lipoprotein cholesterol; ROC, receiver operating characteristic; AUC, operating characteristic curve. **(A)** The four-item factor of symptomatic carotid plaque. **(B)** The ROC curve of the predictive model to identify the symptomatic carotid plaque.

After controlling for potential collinearity and including all possible predictors in the logistic regression analysis, it was found that LDL-C > 3.36 mmol/L (OR, 6.400; 95% confidence interval (CI), 1.067–38.402; *p* = 0.042), irregular plaque (OR, 6.054; 95% CI, 1.016–36.083; *p* = 0.048), ruptured plaque (OR, 6.077; 95% CI, 1.046–35.298; *p* = 0.048), and thrombus (OR, 6.773; 95% CI, 1.194–38.433; *p* = 0.044) were significantly relevant to symptomatic carotid plaque (Table 3). The β-coefficients of the four variables were approximately 1:1:1 (Table 3). The combined predictive model of four independent factors (thrombus, irregular plaque, ruptured plaque, and LDL-C > 3.36 mmol/L) exhibited strong discriminatory ability (area under the curve of 0.924; 95% CI, 0.815–0.979), with a sensitivity of 78.26% and specificity of 93.1% (Figure 2; Table 4).

**Table 3:** Clinical and imaging indicators to identify symptomatic carotid plaque in the final multivariable regression model.

Variable	β-coefficient	SE	Wald	*p-*value	OR	95% CI for OR	
						Lower	Upper
**Clinical indicator**							
LDL-C > 3.36 (mmol/L)	1.856	0.914	4.123	0.042	6.400	1.067	38.402
**DSA indicator**							
Irregular	1.801	0.911	3.909	0.048	6.054	1.016	36.083
**OCT indicator**							
Plaque rupture	1.804	0.898	4.041	0.044	6.077	1.046	35.298
Thrombus	1.913	0.886	4.665	0.031	6.773	1.194	38.433
Constant	−3.703	1.020	13.188	0.000	0.025		

The final multivariable regression model was adjusted for potential collinearity. DSA, digital subtraction angiography; OCT, optical coherence tomography; SE, Standard error; OR, odds ratio; CI, confidence interval.

**Table 4:** ROC curve analysis of combined predictive model and independent risk factors.

	AUC	SE	*Z*	*p*	95%CI	Sensitivity (%)	Specificity (%)
Combined predictive model	0.924	0.035	12.066	0.001	0.815–0.979	78.26	93.10
Thrombus	0.805	0.057	5.387	0.001	0.672–0.902	78.26	82.76
LDL	0.744	0.062	3.928	0.001	0.604–0.855	69.57	79.31
Plaque rupture	0.783	0.059	4.814	0.001	0.647–0.886	73.91	82.76
Irregular	0.736	0.062	3.808	0.001	0.596–0.849	78.26	68.97

The four-item factor of symptomatic carotid plaque and the ROC curve. LDL-C, high-density lipoprotein cholesterol; ROC, receiver operating characteristic; AUC, operating characteristic curve.

## 4 Discussion

The present study describes a combined predictive model with four independent influencing factors, including clinical indicators, angiographical plaque surface morphology assessed by DSA, and plaque characteristics evaluated by OCT to predict the occurrence of symptomatic CAS. Utilizing of multimodality imaging techniques, including the traditional DSA and novel OCT, for carotid plaque assessment facilitates a comprehensive analysis of the risk of plaque. The present research suggests that the four factors, including LDL-C > 3.36 mmol/L, irregular plaque, ruptured plaque, and presence of thrombus, have a potential value when identifying the clinical manifestations of carotid plaque.

OCT is a relatively novel intravascular imaging technique that leverages optical principles with extremely high resolution. OCT has the capability to identify specific characteristics of plaque components, including lipid content, calcification, FCT, plaque rupture, and thrombus, and it can be used to evaluate plaque vulnerability, plaque progression, and drug treatment effects (20–22). It is increasingly becoming the first choice of clinicians due to the ease of image recognition needed to assess carotid artery plaque. Several studies have confirmed that specific characteristics of plaque, like plaque rupture and thrombus, are relevant to the risk of stroke (23, 24).

An OCT-based observational study has shown that vulnerable plaques are more prevalent in symptomatic patients due to their ease of rupture (24). Plaque rupture is characterized by the discontinuity of the fibrous cap and the formation of a clear cavity within the plaque (25). Fibrous cap rupture results in the release of necrotic debris and lipid components from the plaque into the vasculature, where suspension or attachment to the blood vessel wall leads to stroke in the maternal and/or distal vasculature. Plaque rupture was detected on an OCT examination in 17 (79.3%) of 23 patients with symptomatic CAS compared to 5 (17.2%) of 29 patients in the asymptomatic group. Plaque rupture was determined to be the indicator of symptomatic patients, consistent with previous research.

**Figure 3:**
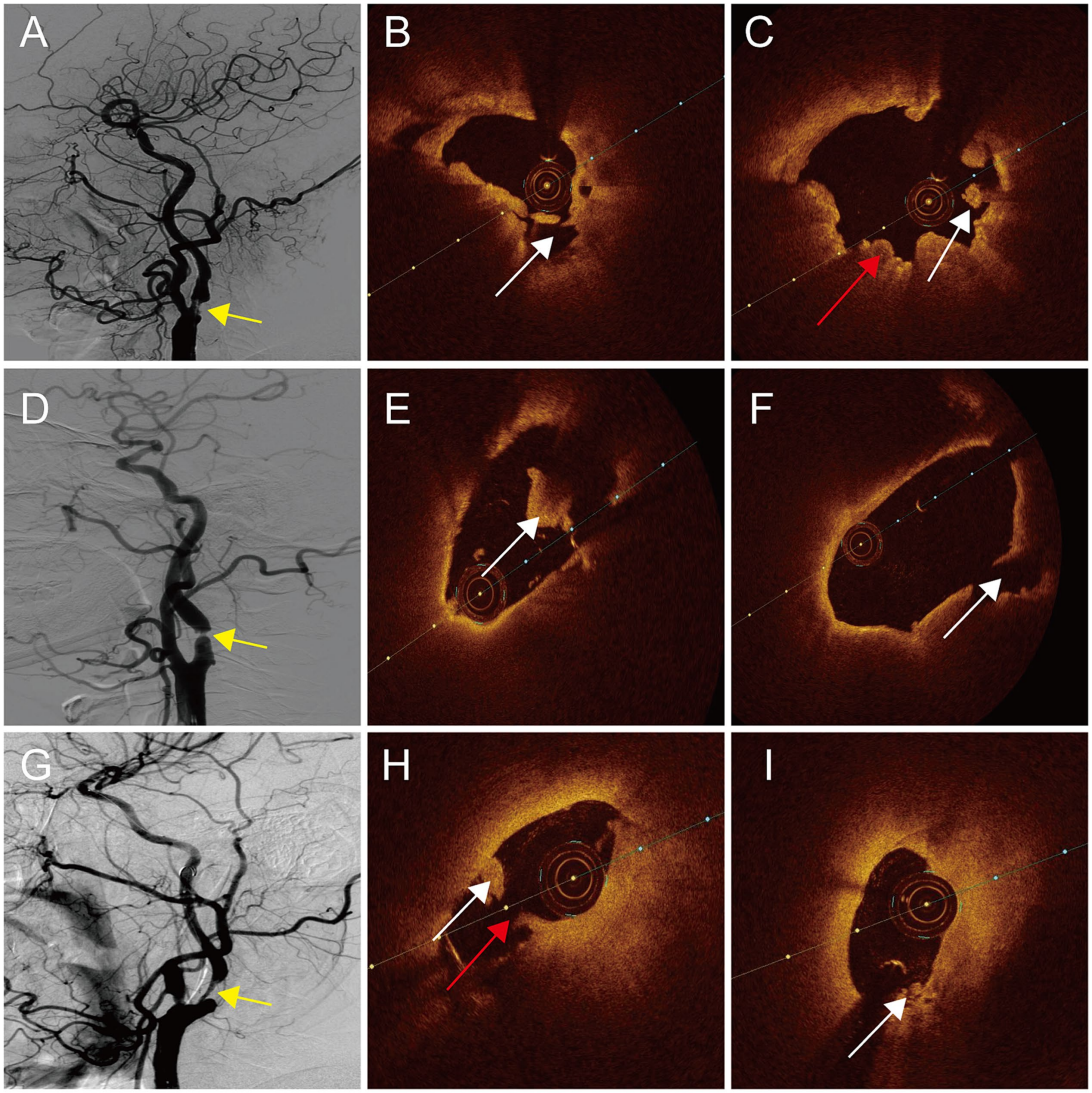
DSA and OCT images of patients with symptomatic carotid atherosclerotic stenosis. **(A,D,G)** Irregular plaques (yellow arrow). **(B)** Plaque rupture of distal (white arrow). **(C)** Red thrombus (red arrow) and white thrombus (white arrow) of proximal. **(E)** White thrombus of distal (white arrow). **(F)** Plaque rupture of proximal (white arrow). **(H)** Plaque rupture (red arrow) and white thrombus (white arrow) of distal. **(I)** Attached white thrombus of proximal (white arrow).

The evaluation of plaque irregularity through DSA has been identified as a significant independent indicator of IS (11). Specifically, carotid plaque surface irregularity, notably plaque ulcers, have been linked to a heightened susceptibility to IS (26). Irregular plaque morphology is considered to be an indicator of symptomatic carotid plaque in the present study. Irregular plaque shown on the DSA image was usually accompanied by plaque rupture, where the formed cavities quickly filled with the contrast agents. Thrombus was observed on a further OCT examination of these irregular plaques. Thrombus recognition on an OCT exam enables to identify mural thrombus that cannot be found using DSA. Red thrombus, white thrombus, and thrombus movement can also be clearly distinguished when the plaque ruptures (10, 27). The presence of a thrombus is a significant element within a complex American Heart Association-VI type plaque (28). There have also been reports suggesting that thrombus is an independent predictor of symptomatic patients (16). In the current investigation, thrombus was determined to be the imaging indicator obtained from OCT necessary for the identification of symptomatic carotid plaque (Figure 3).

Elevated blood cholesterol levels are widely acknowledged as a significant risk factor for the development of ischemic vascular diseases (29). LDL is crucial in the onset and progression of atherosclerotic cardiovascular disease (ASCVD) (30). Consistent association between LDL-C levels and ASCVD risk has been observed, with LDL being considered a conventional biomarker for LDL (31, 32). However, many patients who meet the recommended LDL-C target remain at risk for ASCVD (33). Recently published guidelines for lipid management in Europe and the United States advise to maintain very low LDL-C levels (1.8 mmol/L or less) among individuals with high and very high cardiovascular risk (34). The current study found that a baseline LDL-C level of >3.36 mmol/L was identified as an independent predictor of symptomatic carotid plaque. Previous observational research has demonstrated a significant inverse relationship between HDL-C levels and the risk of ischemic heart disease, with a baseline HDL-C level of <0.925 mmol/L also serving as an independent predictor of symptomatic carotid plaque (29, 35). Additionally, various lipid markers such as HDL-C, TG, TC, and lipoprotein have been linked to atherosclerosis risk, highlighting the critical importance of maintaining optimal blood lipid level (36).

A study of 90 patients with carotid OCT showed that irregular plaque, white thrombus and high-density lipoprotein were risk factors of symptomatic carotid plaque (35). Compared with this study, our study found two new risk factors: low density lipoprotein and plaque rupture, which is a better supplement to the evaluation of the characteristics of symptomatic carotid plaque. But we did not separate the analysis of white thrombus and red thrombus, which is the direction of our further research.

The present study had several advantages, such as utilizing high-resolution OCT imaging data to analyze intricate plaque composition and structure, as well as employing the DSA technique to evaluate plaque surface morphology. The integration of both imaging modalities, each offering unique advantages, results in a more precise and thorough evaluation of plaque characteristics. The present study enrolled patients with DSA-confirmed stenosis atherosclerotic disease (stenosis ≥ 50%). Further research is warranted to investigate the risk predictor markers associated with non-stenotic carotid plaques transitioning to symptomatic status, given their potential contribution to stroke etiology. Therefore, patients with DSA-confirmed non-stenosis atherosclerosis (stenosis < 50%) can be included in further analysis to illustrate clinical and imaging features of symptomatic non-stenosis carotid artery disease.

There were several potential limitations to the present study. Firstly, it was a retrospective single-center study with a relatively limited study population, necessitating further validation of outcomes in a broader cohort. Single-center studies with small sample size inevitably have defects such as selection bias and underrepresentation. As the application of OCT in the cerebrovascular field is still in the exploratory stage, cases are not readily available, especially high-quality OCT images, our model is based on the data currently available at our center. Although the number of cases is limited, the four risk factors identified were more comprehensive and accurate than similar studies. In addition, this is our exploratory study based on a small sample size, hope to share the conclusion with colleagues, also establishes the foundation for future multi-center research. With the increase of the sample size, we will verify and revise the stability and validity of the model by large sample size and multiple centers. Secondly, unavoidable selection bias may have been introduced by the exclusion of patients with tight stenosis, severe tortuosity, or suboptimal luminal blood clearance in the target carotid arteries. Thirdly, the potential underestimation of cholesterol crystals may be attributed to factors such as the substantial lipid pool, intraluminal thrombus, or restricted penetration of OCT. Fourth, qualitative plaque composition was not assessed using OCT. Finally, long-term follow-up data following the intervention were not provided, prompting the need for further analysis of stroke recurrence in patients with symptomatic carotid plaque and the initial occurrence of stroke in asymptomatic patients in future studies.

## 5 Conclusion

The integration of LDL-C, irregular plaque, ruptured plaque, and thrombus into a predictive model has the potential to identify symptomatic carotid plaque. The subsequent phase of this study will be to explore the utilization of the model to inform the development of personalized optimal treatment strategies (including optimal drug therapy, carotid stenting, carotid endarterectomy), and to validate its clinical utility through follow-up.

## Data Availability

The raw data supporting the conclusions of this article will be made available by the authors, without undue reservation.
